# iDESC: identifying differential expression in single-cell RNA sequencing data with multiple subjects

**DOI:** 10.1186/s12859-023-05432-8

**Published:** 2023-08-22

**Authors:** Yunqing Liu, Jiayi Zhao, Taylor S. Adams, Ningya Wang, Jonas C. Schupp, Weimiao Wu, John E. McDonough, Geoffrey L. Chupp, Naftali Kaminski, Zuoheng Wang, Xiting Yan

**Affiliations:** 1 Department of Biostatistics, Yale School of Public Health, New Haven, CT 06520 USA; 2 Section of Pulmonary, Critical Care and Sleep Medicine, Yale School of Medicine, New Haven, CT 06520 USA; 3 Department of Respiratory Medicine, Hannover Medical School and Biomedical Research in End-Stage and Obstructive Lung Disease Hannover, German Center for Lung Research (DZL), Hannover, Germany; 4 Meta Platforms, Inc, Cambridge, USA

**Keywords:** Single-cell RNA sequencing, Differential expression analysis, Subject effect, Zero-inflated negative binomial mixed model

## Abstract

**Background:**

Single-cell RNA sequencing (scRNA-seq) technology has enabled assessment of transcriptome-wide changes at single-cell resolution. Due to the heterogeneity in environmental exposure and genetic background across subjects, subject effect contributes to the major source of variation in scRNA-seq data with multiple subjects, which severely confounds cell type specific differential expression (DE) analysis. Moreover, dropout events are prevalent in scRNA-seq data, leading to excessive number of zeroes in the data, which further aggravates the challenge in DE analysis.

**Results:**

We developed iDESC to detect cell type specific DE genes between two groups of subjects in scRNA-seq data. iDESC uses a zero-inflated negative binomial mixed model to consider both subject effect and dropouts. The prevalence of dropout events (dropout rate) was demonstrated to be dependent on gene expression level, which is modeled by pooling information across genes. Subject effect is modeled as a random effect in the log-mean of the negative binomial component. We evaluated and compared the performance of iDESC with eleven existing DE analysis methods. Using simulated data, we demonstrated that iDESC had well-controlled type I error and higher power compared to the existing methods. Applications of those methods with well-controlled type I error to three real scRNA-seq datasets from the same tissue and disease showed that the results of iDESC achieved the best consistency between datasets and the best disease relevance.

**Conclusions:**

iDESC was able to achieve more accurate and robust DE analysis results by separating subject effect from disease effect with consideration of dropouts to identify DE genes, suggesting the importance of considering subject effect and dropouts in the DE analysis of scRNA-seq data with multiple subjects.

**Supplementary Information:**

The online version contains supplementary material available at 10.1186/s12859-023-05432-8.

## Background

Recent advances in droplet-based single-cell RNA sequencing (scRNA-seq) technology have enabled investigators to assess transcriptome-wide differences at single-cell resolution [1–3]. Instead of pooling RNAs from all cells together, droplet-based scRNA-seq technology isolates cells using oil droplets, in which each cell is lysed and a cell barcode and a unique molecular identifier (UMI) are added onto the amplified cDNAs. Using these cell barcodes and UMIs, sequencing reads are demultiplexed into different cells and transcripts, which enables single-cell transcriptome profiling without PCR amplification bias. In recent years, scRNA-seq has been used to study cellular heterogeneity and gene expression variability across different cell types in diverse human tissues [4] and diseases (chronic diseases [5], infectious diseases [6], autoimmune diseases [7], and cancers [8]). These applications have revealed disease-related cell type specific transcriptomic changes [9], rare cell types [10], and cell type composition changes [11], providing important insights into disease pathogenesis and facilitating the development of personalized treatment of diseases [12, 13].

Despite the great potential of scRNA-seq technology, challenges remain in the corresponding data analysis. Specifically, one common task in scRNA-seq data analysis is to identify cell type specific differentially expressed (DE) genes between two groups of subjects [14], which can be challenging due to prevalent dropout events and substantial subject effect, or so-called between biological replicate variation [15]. Dropout refers to the event when a given gene is observed at a moderate expression level in one cell but is not detected in another cell of the same type from the same sample [16], leading to underestimation of gene expression level and overestimation of variation in the data which may generate false positive results. Moreover, with the popularity of multi-sample scRNA-seq datasets from different diseases, tissues, and cell types, many of them have consistently shown that within the same cell type, cells of the same subject cluster together but separate well from cells of other subjects [6, 17, 18]. This suggests that there exists a large variation across subjects possibly due to heterogeneous genetic backgrounds or environmental exposures and this variation is much larger than the within-subject variation across cells of the same type. Dominant subject effect severely confounds the DE analysis of scRNA-seq data because genes driving differences across subjects are likely to also be significantly different between two groups [15, 19, 20]. Taken together, in the DE analysis of scRNA-seq data with multiple subjects, it is critical to separate subject effect from disease effect with consideration of dropout events.

Technical batch effect is one possible reason for the large variation across subjects because many studies processed cells and cDNA libraries from different subjects in different batches due to the requirement of sample freshness in certain tissue types and early-stage scRNA-seq protocols. This may lead to batch effect in the data so that cells of the same type from different subjects have different expression profiles. However, in the scRNA-seq dataset from patients with idiopathic pulmonary fibrosis (IPF), the large variation across subjects was still present after adjusting for batch effect using scVI [21]. In addition, recent advances in combining scRNA-seq with upstream cell cryopreservation using dimethyl sulfoxide (DMSO) have enabled preservation of cells so that samples from different subjects can be processed together [22]. Comparison between scRNA-seq data from the same sputum sample with and without DMSO preservation showed no significant difference between the fresh and DMSO data but significant separation between different subjects was still present (unpublished data). Since the fresh and DMSO data from the same sample were generated in two different batches, this confirmed that the large between-subject variation was a consequence of biological subject effect but not technical batch effect. Therefore, it is inadequate to consider this variation as technical batch effect and remove it using batch effect adjustment tools in scRNA-seq data. In fact, removing this variation as technical batch effect may remove the disease effect of interest because subject effect confounds with disease effect.

Many DE analysis methods for scRNA-seq data have been developed and compared [23–25]. They can be classified into two categories depending on whether subject effect is considered. Although methods that ignore subject effect have been used in DE analysis, they are more suitable for identification of marker genes for a given cell type, which is fundamentally different from DE analysis.

Within the category of methods that ignore subject effect, there are methods specifically designed for scRNA-seq data and methods adopted from bulk RNA-seq DE analysis. Among the methods designed for scRNA-seq data, BASiCS [26] and TASC [27] require external RNA spike-ins to provide information on technical variation and use a Bayesian hierarchical Poisson-Gamma model and a hierarchical Poisson-lognormal model, respectively, to fit data. Monocle [28–30] and NBID [31] model UMI counts of each gene using a negative binomial distribution without considering dropouts. To account for dropouts, a group of methods were developed including DEsingle [32], DESCENT [33], SC2P [34], SCDE [16] and MAST [35]. These methods utilize mixture models or hierarchical models, mostly zero-inflated, to model dropouts and captured transcripts. DEsingle fits a zero-inflated negative binomial model in each group and conducts a likelihood ratio test for significance assessment. DESCENT models UMI counts using a hierarchical model which assumes that the true underlying expression follows a zero-inflated negative binomial distribution and the capturing process generating the observed data follows a beta-binomial distribution. SC2P models dropout events using a zero-inflated Poisson distribution and fits the detected transcripts using a lognormal-Poisson distribution. The assumption in SC2P that the cell-specific dropout rate and dropout distribution are shared by all genes may eliminate the natural stochasticity in scRNA-seq data. SCDE employs a two-component mixture model with a negative binomial and a low-magnitude Poisson component to model efficiently amplified read-outs and dropout events, respectively. The dropout rate for a given gene is determined by its true underlying expression level in the cell, which is estimated based on a selected subset of highly expressed genes. MAST uses a two-part hurdle model in which dropout rates are modeled by a logistic regression model and non-zero expression follows a Gaussian distribution. SC2P, SCDE and MAST were originally designed for Transcript Per Kilobase Million (TPM) data which has different technical noise and data distribution from UMI count data [36]. Multimodality has been observed in scRNA-seq data due to cellular heterogeneity within the same cell type. To consider multimodality, scDD [37] was designed to model count data with a Dirichlet process to detect genes with difference in mean expression, proportion of the same component, or modality between groups. D3E [38], a nonparametric method, fits a bursting model for transcriptional regulation and compares the gene expression distribution between two groups. It was previously reported to generate false-positive results on negative control datasets [24]. A recent study [23] showed that bulk RNA-seq analysis methods, including DESeq2 [39], limma-trend [40], and Wilcoxon rank sum test [41], have comparable performance to methods designed for scRNA-seq data when applied to the cell-level UMI count data, especially after filtering out lowly expressed genes.

All the methods mentioned above treat cells from the same subject as independent, which may be efficient for identifying cell type marker genes, but inappropriate for DE analysis to identify disease or phenotype associated genes due to the presence of dominant subject effect confounded with disease effect as described above. One simple and straightforward solution is to aggregate expression levels of cells from the same subject by averaging and then to compare the aggregated sample-level “pseudo-bulk” expression levels between two groups of subjects using Student’s t test. We denote this method as subject-t-test (subT). Furthermore, two recent studies proposed the following three DE analysis methods to consider subject effect in scRNA-seq data. Zimmerman et al. [19] developed MAST-RE by adding a subject random effect to the non-zero expression part of the hurdle model in MAST. The muscat package [20] provides two approaches to consider subject effect: (1) muscat-PB that aggregates cell-level UMI counts into sample-level “pseudo-bulk” counts which are then compared between two groups using edgeR that was developed for DE analysis in bulk RNA-seq data; and (2) muscat-MM that fits a generalized linear mixed model (GLMM) on the cell-level UMI counts to account for subject variation. Both muscat-PB and muscat-MM were compared to other methods and shown to have power gain by considering subject effect [15].

In this article, we develop a new statistical model to identify DE genes in scRNA-seq data with multiple subjects, named iDESC. A zero-inflated negative binomial mixed model is used to consider both subject effect and dropouts. iDESC models dropout events as inflated zeros by pooling information across genes and assuming that genes with similar expression share similar dropout rates. In addition, iDESC allows dropout rate to be subject/batch specific. The non-dropout events are modeled by a negative binomial distribution. In the negative binomial component, a random effect is used to separate subject effect from disease effect. Wald statistic is used to assess the significance of disease effect. We compared iDESC with 11 existing DE analysis methods based on type I error, statistical power, between-dataset consistency and validation using both simulated and real datasets.

## Results

### Dependency of dropout rate on gene expression

**Fig. 1:**
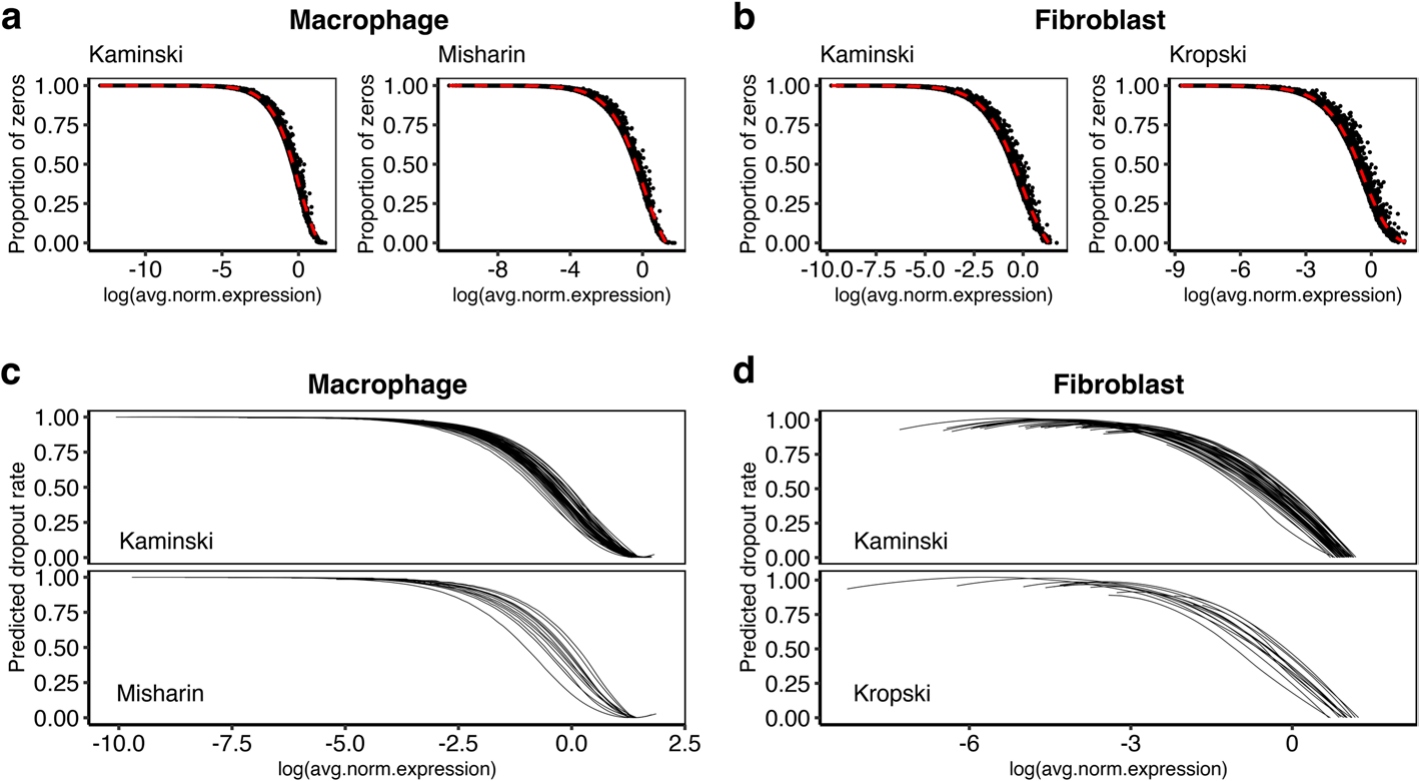
Dependency of dropout rate on gene expression. Plots showing the gene-level proportion of zeros in relation to the average log-normalize UMI count across all cells in **a** the Kaminski and Misharin macrophage datasets and **b** the Kaminski and Kropski fibroblast datasets. The LOESS curves (red lines) described decreasing patterns of dropout rate on gene expression level. The subject-level LOESS curves in **c** the Kaminski and Misharin macrophage datasets and **d** the Kaminski and Kropski fibroblast datasets demonstrated heterogeneity of this dependency across different subjects

Previous studies reported that the dropout rate of a gene in a given cell depends on the expected expression level of the gene in the cell and dropout events are more prevalent for genes with lower expression [16]. As expected, in both macrophage and fibroblast across the three real datasets, we observed decreasing patterns in the gene-level proportion of zeros when the average log-normalized UMI count across all cells increases (Fig. 1a, b). Assuming that genes with similar average expression share similar dropout rates, we pooled information across genes to obtain an S-shape curve describing the dependency of dropout rate on gene expression level using locally estimated scatterplot smoothing (LOESS) regression. Heterogeneity of this dependency across different subjects was also observed when we stratified cells by subjects to obtain the subject-level LOESS curves, suggesting that dropout rate is likely to be subject/batch specific (Fig. 1c, d).

### Method performance evaluation overview

We evaluated and compared the performance of iDESC and eleven existing DE analysis methods (Table 1) using both simulation studies and three real datasets. We divide these methods into two categories, based on whether subject effect is considered. The first category of methods considers subject effect, including iDESC, MAST-RE, muscat-PB, muscat-MM and subT. Among them, iDESC, MAST-RE and muscat-MM are mixed model-based methods, whereas muscat-PB and subT are aggregation-based methods. iDESC and MAST-RE also consider dropouts in scRNA-seq data. The other category of methods does not consider subject effect. Within this category, DEsingle, MAST and scDD consider dropouts while NBID, DESeq2, limma-trend and Wilcoxon rank sum test do not. DEsingle considers dropout in the fitted model but tests for differences in both group means and dropout rates instead of difference in group means only. Therefore, unlike most DE methods, DEsingle identifies genes with composite differences in group means and dropout rates. Comparison of these methods enabled us to assess the benefit of considering dropout evens and/or subject effect in the DE analysis of scRNA-seq data.

**Table 1:** Overview of the twelve DE analysis methods for comparison

	Dropout	Subject effect	Test	Model
iDESC	✓	Mixed model	Wald test	Zero-inflated negative binomial mixed model
MAST-RE	✓	Mixed model	Likelihood ratio test	Two-part hurdle mixed model
Muscat-MM	×	Mixed model	Wald test	Negative binomial mixed model
Muscat-PB	×	Aggregation	Quasi-likelihood F-test	EdgeR on sample-level aggregated data
subT	×	Aggregation	Student’s T test	T test on sample-level aggregated data
DEsingle	✓	×	Likelihood ratio test	Group-specific zero-inflated negative binomial model
MAST	✓	×	Likelihood ratio test	Two-part hurdle model
scDD	✓	×	Kolmogorov–Smirnov test	Dirichlet process mixture of normals
NBID	×	×	Likelihood ratio test	Negative binomial model with group-specific dispersion
DESeq2	×	×	Wald test	Negative binomial model with the same dispersion between groups
limma	×	×	Moderated T test	Linear regression model
Wilcoxon	×	×	Wilcoxon rank sum test	Nonparametric test

We compared method performance based on type I error, statistical power, and consistency of the identified DE genes in three independent scRNA-seq datasets on the same disease, the Kaminski dataset [18], the Kropski dataset [42] and the Misharin dataset [17]. All datasets measured scRNA-seq data of whole lung tissue from patients with IPF and normal controls.

### Type I error assessment

**Fig. 2:**
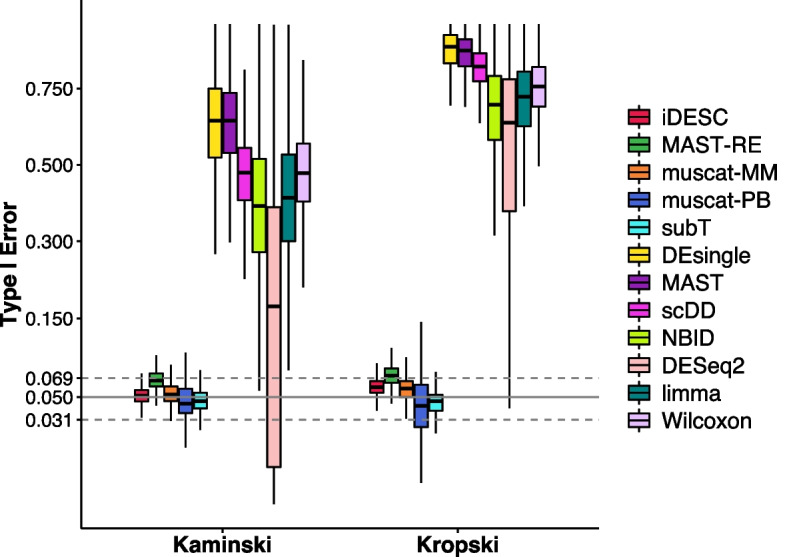
Empirical type I error of all methods on the two permuted real datasets. Boxplots showing the median (center line), interquartile range (hinges), and 1.5 times the interquartile (whiskers) of empirical type I error at the nominal level of 0.05. Confidence interval of type I error is marked by two dashed lines (0.031–0.069)

To assess type I error, we permuted the group labels of subjects in both Kaminski and Kropski datasets 500 times. All twelve methods were applied to the permuted datasets to identify DE genes in macrophages. For each gene, we calculated the empirical type I error as the proportion of permuted datasets with a *p* value < 0.05 and compared it to the nominal level 0.05. Figure 2 shows the empirical type I error of each method. In both datasets, methods that account for subject effect, including iDESC, MAST-RE, muscat-MM, muscat-PB and subT, had well-controlled type I error. Among these methods, MAST-RE had slightly inflated type I error for some genes, likely due to the deviation of log-normalized UMI counts from the assumed Gaussian distribution. In contrast, the type I error of the methods that do not consider subject effect were severely inflated. The inflation of type I error was more prominent in the Kropski dataset for these methods, indicating a larger subject effect in the data. Among these methods, DEsingle, MAST and scDD had the largest inflation in type I error. DESeq2 had the largest variation in type I error across all genes. Taken together, these results suggested that it is important to consider subject effect for type I error control in the DE analysis of scRNA-seq data with multiple subjects.

### Power comparison

To compare power, we simulated scRNA-seq data with 150 true DE genes and 300 non-DE genes under a wide range of parameter settings estimated from the Kaminski macrophage dataset. Expression data of the DE genes was simulated to have a fold-change of ${e}^{\beta }$ between the two groups of subjects. The number of cells per subject ($m$), the magnitude of capture efficiency ($\delta$) and the log fold change ($\beta$) were varied at different levels to simulate different datasets. We then applied all twelve methods to each simulated dataset for DE analysis. Method performance was assessed by the area under a receiver operating characteristic curve (AUC) that describes the sensitivity and specificity of the identified DE genes under different significance levels. The sensitivity and specificity under the p-value threshold of 0.05 are also demonstrated.

**Fig. 3:**
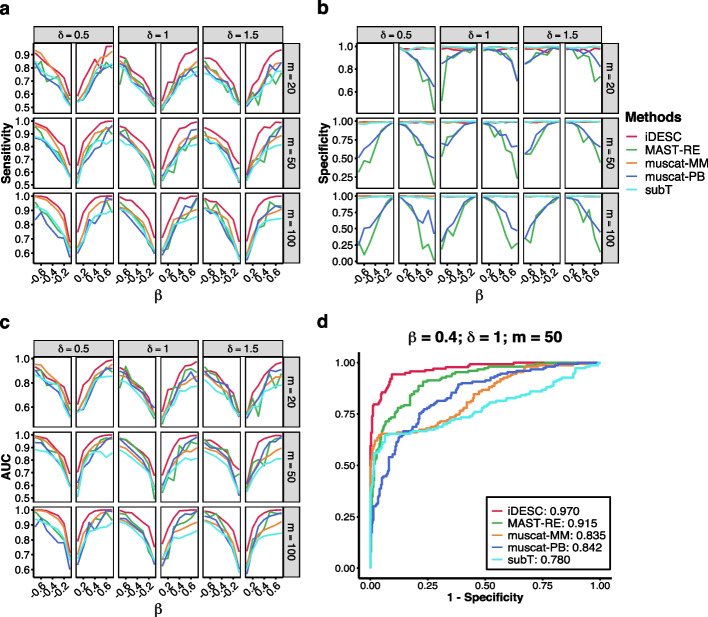
Power comparison of methods considering subject effect in simulated datasets. Evaluation criteria including **a** sensitivity and **b** specificity under the p-value threshold of 0.05, and **c** area under an ROC curve (AUC) to measure the accuracy of identified DE genes under three levels of capture efficiency (δ) and number of cells per subject ($m$). **d** ROC curves and the corresponding AUC scores when $\beta =0.4, \delta =1, m=50$

Figures 3 and Additional file 1: S1 show that all methods had improved sensitivity and AUC when the number of cells per subject increased except scDD. Under most simulation settings, iDESC performed the best with the highest sensitivity and AUC when compared to methods considering subject effect (Fig. 3a, c, d). In the setting with 20 cells per subject and negative group effect, iDESC had comparable or higher AUC than other methods. When capture efficiency was high ($\delta =0.5$) corresponding to low dropout rate, muscat-MM had the second highest sensitivity and AUC but was better than iDESC in the setting with 20 cells per subject and negative disease effect. When capture efficiency was low ($\delta =1, 1.5$) corresponding to high dropout rate, the other four methods that consider subject effect, MAST-RE, muscat-MM, muscat-PB and subT, were comparable and had lower sensitivity and AUC than iDESC. The specificity of iDESC, muscat-MM and sub-T were around 0.95 at the p-value threshold of 0.05, while MAST-RE and muscat-PB had lower specificity especially when the group effect was large (Fig. 3b). When we compared iDESC with the other seven methods that do not consider subject effect, DEseq2 had comparable or even higher sensitivity and AUC than iDESC when capture efficiency was low ($\delta =1, 1.5$), but performed worse when capture efficiency was high ($\delta =0.5$) (Additional file 1: Figs. S1a, S1c and S1d). DEseq2 and Wilcoxon had lower specificity especially when capture efficiency was high ($\delta =0.5$) (Additional file 1: Fig. S1b). All other methods had lower sensitivity, specificity and AUC. scDD and NBID had compromised performance in most of the simulation settings. In summary, iDESC had comparable or the highest sensitivity and AUC, and maintained the correct specificity across all simulation settings.

### Consistency and validation of results in three independent scRNA-seq datasets

**Fig. 4:**
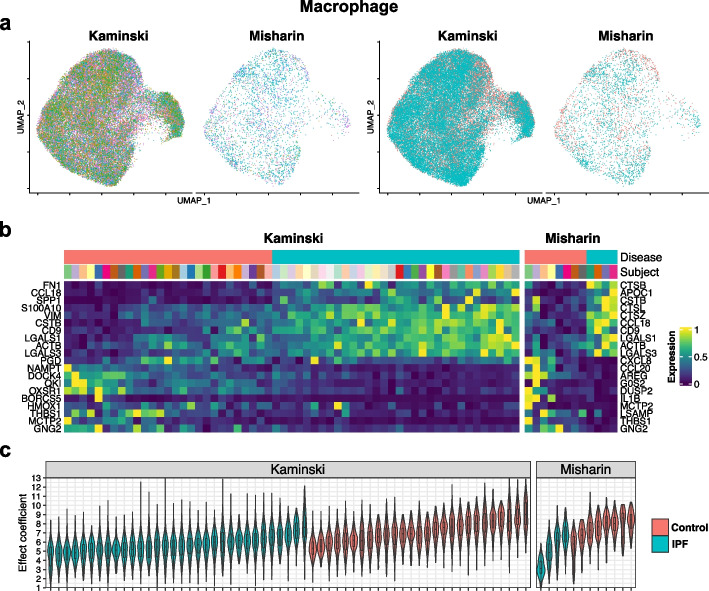
DE analysis using iDESC on the two IPF macrophage datasets. **a** UMAP of nomenclature matched macrophages in the Kaminski and Misharin datasets colored by subject (left) and group (right). **b** Heatmap of subject-level average expression for the top 10 upregulated and top 10 downregulated genes. **c** Violin plots demonstrate cell-level contributions to the group fold-change within each subject. Each violin corresponds to one subject and is colored by group

**Fig. 5:**
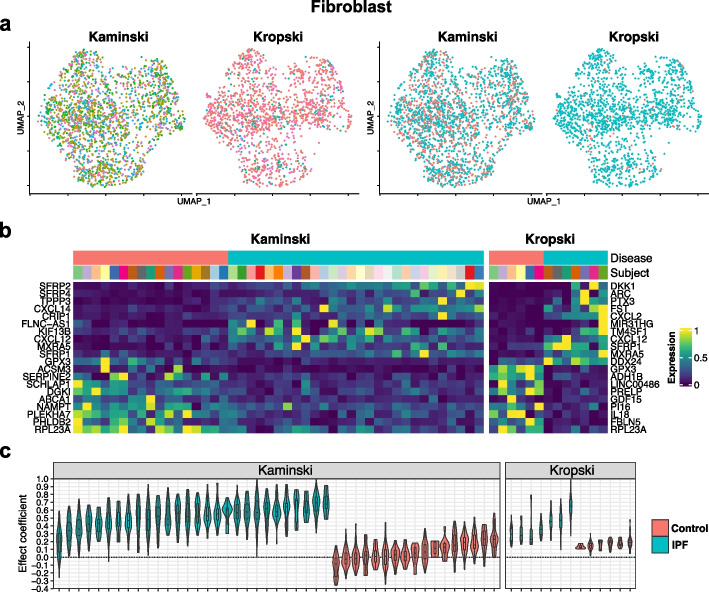
DE analysis using iDESC on the two IPF fibroblast datasets. **a** UMAP of nomenclature matched fibroblasts in the Kaminski and Kropski datasets colored by subject (left) and group (right). **b** Heatmap of subject-level average expression for the top 10 upregulated and top 10 downregulated genes. **c** Violin plots demonstrate cell-level contributions to the group fold-change within each subject. Each violin corresponds to one subject and is colored by group

We used two cell types, macrophage and fibroblast, from three independent scRNA-seq datasets (Kaminski, Kropski and Misharin) of whole lung tissue from IPF patients and normal controls [17, 18, 42] to demonstrate and compare the between-dataset consistency of DE results by different methods. Both cell types have been reported to have significant transcriptomic changes in IPF patients [43–50]. Since different datasets have different cell type nomenclature, we overlaid the data from all three datasets to find subpopulations of cells that overlap well across the three datasets on the UMAP of integrated data by Seurat (Figs. 4a and 5a). This step was performed to ensure that DE analysis was conducted on the same type of cells across three datasets so that results are comparable. For each cell type, we selected datasets with a median number of cells per subject larger than 10 to conduct the consistency analysis. Eventually, the DE analysis results in macrophage were compared between the Kaminski and Misharin datasets and in fibroblast between the Kaminski and Kropski datasets. Five methods that consider subject effect, including iDESC, MAST-RE, muscat-MM, muscat-PB and subT, were applied. After data preprocessing, we had 7,128 genes in 43,028 macrophages from the Kaminski dataset, and 8,409 genes in 3,635 macrophages from the Misharin dataset. For fibroblast, we had 10,860 genes in 2,290 fibroblasts from the Kaminski dataset, and 9,325 genes in 1,615 fibroblasts from the Kropski dataset. We further had 7,653 genes in 8,663 macrophages from the Kropski dataset for external validation.

At the threshold of *p* value < 0.01, iDESC identified 5,577 and 1,124 DE genes in Kaminski and Misharin macrophage datasets, respectively, and 417 and 534 DE genes in Kaminski and Kropski fibroblast datasets, respectively. The top upregulated DE genes in IPF macrophage (Fig. 4b), such as *FN*1, *CCL*18 and *SPP*1, were previously reported to be upregulated in IPF and related to IPF pathogenesis in macrophages [17, 18, 44, 45, 47, 48, 51]. In fibroblast, *CXCL*14 and *SFRP*1 were identified among the top upregulated DE genes in IPF (Fig. 5b), which were also found to be potential signatures of IPF in previous studies [49, 50, 52–54]. iDESC also identified *CXCL*12, a gene potentially related to the pulmonary fibrosis progression [55, 56], to be upregulated in IPF fibroblast. To examine subject variations in these three datasets, we calculated cell-level effect coefficients [20] for each subject in macrophage (Fig. 4c) and fibroblast (Fig. 5c). Cell-level effect coefficients summarized the extent to which each cell reflects the group-level fold-change. For each cell type, both inter- and intra-subject variations of effect coefficients are different between the two chosen datasets, suggesting that the level of subject variation varies across different datasets potentially due to variations in biological background of subjects.

**Fig. 6:**
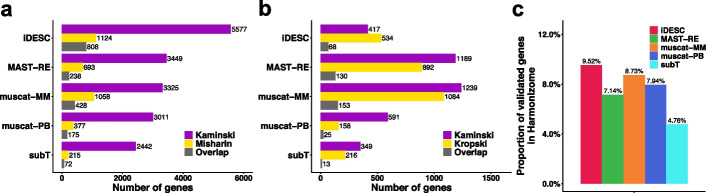
Consistency and validation of DE genes overlapping between real datasets in macrophage and fibroblast. **a** Barplots showing the number of DE genes identified in the Kaminski (purple), Misharin (yellow) datasets and the overlap (grey) between them in macrophage. **b** Barplots showing the number of DE genes identified in the Kaminski (purple), Kropski (yellow) datasets and the overlap (grey) between them in fibroblast. **c** Barplots showing the percentage of IPF-related genes in the Harmonizome database identified in both the Kaminski and Misharin macrophage datasets

We further evaluated method performance based on the consistency of DE genes between datasets. For each method in each cell type, we overlapped the identified DE genes from the two chosen datasets (Fig. 6a, b). Fisher’s exact test was conducted to assess the significance of overlap and Jaccard index was calculated to measure the similarity between the two DE gene lists for each method (Table 2). The higher the overlap is, the more consistent the results are between datasets, indicating a better performance. Figure 6a shows that iDESC identified the largest number of between-dataset overlapping DE genes (808 genes) in macrophage. Although iDESC did not achieve the most between-dataset overlapping DE genes in fibroblast (Fig. 6b), Table 2 shows that iDESC had the most significant between-dataset overlap in both cell types (macrophage: *p* = 1 × 10^–5^, fibroblast: *p* = 4 × 10^–21^), followed by muscat-PB (macrophage: *p* = 1 × 10^–5^, fibroblast: *p* = 2 × 10^–7^). In contrast, MAST-RE, muscat-MM and subT did not achieve significant between-dataset overlap in macrophage. They had significant overlap in fibroblast while their p-values were much larger than iDESC and others. In addition, iDESC had the largest Jaccard Index in both cell types (macrophage: JI = 0.137, fibroblast: JI = 0.077), followed by muscat-MM (macrophage: JI = 0.108, fibroblast: JI = 0.071).

**Table 2:** Fisher’s exact test and Jaccard index measuring the DE genes overlapping between the two chosen scRNA-seq datasets in macrophage and fibroblast

Method		iDESC	MAST-RE	Muscat-MM	Muscat-PB	subT
Macrophage	*P* value	$1\times {10}^{-5}$	$1.000$	$0.269$	$1\times {10}^{-5}$	0.085
	Jaccard Index	0.137	$0.061$	$0.108$	0.055	0.028
Fibroblast	*P* value	$4\times {10}^{-21}$	$4\times {10}^{-6}$	$3\times {10}^{-5}$	$2\times {10}^{-7}$	0.011
	Jaccard Index	0.077	0.067	0.071	0.035	0.024

Lastly, we used a list of 83 IPF-related genes in the Harmonizome database [57] to validate the between-dataset overlapping DE genes in each cell type identified by each method (Fig. 6c). In macrophage, iDESC had the highest proportion of validated genes (9.52%), followed by muscat-MM (8.73%), muscat-PB (7.94%), MAST-RE (7.14%) and subT (4.76%). In fibroblast, none of the methods had more than 3 genes that were found in the Harmonizome gene list, thus the validation results were not suitable for comparison.

To validate the top DE genes identified in the Kaminski macrophage dataset, we used the Kropski and Misharin macrophage datasets. Table 3 demonstrates that the top 10 upregulated DE genes identified using iDESC in the Kaminski macrophage dataset, if captured in either of the two validation datasets, were also significant (*p* < 0.01) by iDESC in the Kropski and Misharin macrophage datasets. We also examined the expression distribution of the top 3 DE genes, *FN*1, *CCL*18 and *SPP*1, identified in the Kaminski macrophage dataset, in the three IPF macrophage datasets. Additional file 2: Figure S2 shows that all three genes were differentially expressed between IPF and control (*p* < 0.01, Wilcoxon rank sum test) in the three IPF macrophage datasets.

**Table 3:** DE analysis results using iDESC of the top 10 up-regulated DE genes, identified in the Kaminski macrophage dataset, in the three IPF macrophage datasets

Gene	Kaminski		Kropski		Misharin	
	Fold change	*P* value	Fold change	*P* value	Fold change	*P* value
*FN1*	2.882	7.01×10^−32^	2.639	3.69×10^−10^	*NA*	*NA*
*CCL18*	2.528	3.95×10^−22^	2.594	3.68×10^−4^	1.725	5.79×10^−4^
*SPP1*	2.201	6.39×10^−13^	2.444	1.63×10^−3^	*NA*	*NA*
*S100A10*	1.827	2.75×10^−17^	2.418	3.27×10^−4^	*NA*	*NA*
*VIM*	1.807	1.19×10^−29^	*NA*	*NA*	1.392	1.14×10^−3^
*CSTB*	1.777	2.79×10^−21^	2.247	3.11×10^−8^	2.106	2.35×10^−3^
*CD9*	1.702	2.71×10^−17^	1.517	8.90×10^−3^	1.677	2.44×10^−3^
*LGALS1*	1.690	8.79×10^−11^	2.033	2.22×10^−6^	1.633	9.19×10^−4^
*ACTB*	1.568	6.02×10^−9^	*NA*	*NA*	1.597	7.35×10^−3^
*LGALS3*	1.508	1.00×10^−13^	1.929	1.83×10^−3^	1.530	4.30×10^−3^

NA represents that the gene was filtered and not included in the DE analysis in the corresponding dataset

In summary, we evaluated method performance using real datasets based on consistency of results across different datasets and validation using previously reported IPF associated genes in public database and literatures. Our method, iDESC, achieved the best performance based on both evaluation criteria. The top DE genes identified by iDESC were highly biologically relevant, well supported by literatures, and validated by two other independent datasets.

### Computation time

The runtime of iDESC is 33.3 and 24.6 min to analyze the Kaminski and Kropski fibroblast datasets, respectively, using a 10-core, 100 GB RAM, Intel Xeon 2.6 GHz CPU machine. The computation time of iDESC is relatively long because of the mixed model fitting for subject effect and the consideration of dropouts. For a dataset of 50–100 subjects and ~ 2000 cells per sample, iDESC took about 50 h using a 10-core, 100 GB RAM, Intel Xeon 2.6 GHz CPU machine. To provide more information on computation time, we recorded the time of the five methods that consider subject effect, iDESC, MAST-RE, muscat-MM, muscat-PB and subT, on 10 genes in the Kaminski fibroblast dataset that includes 2,290 fibroblasts. Additional file 3: Table S1 displays the runtime on a single core. With parallel computing, the runtime of the mixed model-based methods, iDESC and muscat-MM, was further reduced to 9.74 and 9.51 s, respectively, on a 10-core machine.

## Discussion

We have developed a new method, iDESC, to detect cell type specific DE genes between two groups of subjects in scRNA-seq data. iDESC fits a zero-inflated GLMM assuming dropouts to have zero count and captured expression to have a negative binomial distribution. Information across genes were pooled to model the dependency of dropout rate on gene expression level. Subject effect is modeled as a random effect in the log-mean of the negative binomial component. Wald test is used to assess the group mean difference in captured transcripts. We compared the performance of iDESC with elevent exiting DE analysis methods using both simulated data and real datasets. Permutation analysis using real data demonstrated that the type I errors of methods that consider subject effect were well calibrated, whereas the type I errors of methods that ignore subject effect were highly inflated. Using simulated data based on parameters estimated from real datasets, we showed that iDESC achieved comparable or higher power among methods that consider subject effect. In three independent scRNA-seq datasets of IPF patients and healthy controls, several of the top DE genes identified by iDESC were well supported by literatures regarding their important roles in IPF pathogenesis. Moreover, iDESC achieved the most consistent and validated results between independent datasets and using public database, respectively. These results demonstrated superior performance of iDESC over the other existing methods, suggesting the importance of considering subject effect and dropouts in the DE analysis of scRNA-seq data with multiple subjects.

Like most DE analysis methods for scRNA-seq data, iDESC requires accurate cell type annotation, which is a key step to ensure the validity and biological relevance of the downstream DE analysis. Cell clustering and cell type annotation are commonly performed with the removal of technical (batch) or biological (subject) effects through data integration. During this step, group/disease effect will be removed along with subject effect. Therefore, the downstream DE analysis will be performed on the original count or normalized data in each annotated cell type instead of the integrated data. Inaccurate cell typing may lead to data distribution deviated from the negative binomial distribution or with multiple modes. Possible remedies to reduce the negative impact of inaccurate cell type annotation include the following strategies. First, we strongly recommend examining the cell distribution through data visualization using UMAP and/or t-SNE plots and performing cell clustering to detect potential incorrect cell annotations. Second, a goodness-of-fit test for iDESC and/or replacing the negative binomial distribution with a multi-modal distribution or a mixture model may improve model fitting.

Despite the advantages of iDESC over the other DE analysis methods shown in this article, iDESC can be improved in several directions. First, in some cell types, when the cell-to-cell heterogeneity of certain genes is high or heterogeneous cell subtypes exist, negative binomial distribution may not fit the data well. Especially when heterogeneous cell subtypes are present in the data, the distribution of expression may be multi-modal. Data transformation, a goodness-of-fit test for iDESC and/or replacing negative binomial distribution with a multi-modal distribution or a mixture model may improve the model fitting. Second, estimation of dispersion parameter in a negative binomial distribution has been shown to be challenging. Multiple dispersion correction approaches [39, 58–60] that have been developed to improve accuracy can be used to further improve the performance of iDESC.

The computational speed of iDESC is relatively slow due to its consideration of dropouts and subject effect, which are critical for iDESC to achieve significant improvement in performance. Besides implementing parallel computing on high performance computers, the following two future work can potentially reduce the runtime of iDESC. First, the “glmmTMB” package uses the Template Model Builder (TMB) framework to calculate the first and second order derivatives of the likelihood function by automatic differentiation (AD). It is possible to speed up the algorithm by specifying the calculation of first and second order derivatives for the quasi-likelihood function of our model to skip this process. Second, the objective function of iDESC was optimized using the "nlminb" optimizer in an iterative scheme, which is an unconstrained quasi-Newton method optimizer. Replacing nlminb with a more efficient algorithm such as stochastic gradient descent (SGD) may further reduce the runtime.

## Conclusions

We developed iDESC, a zero-inflated negative binomial mixed model that considers both subject effect and dropouts, to identify cell type specific differentially expressed genes in scRNA-seq data with multiple subjects. iDESC had well-calibrated type I error and comparable or higher power than other existing DE methods. When applied to three independent scRNA-seq datasets with IPF patients and healthy controls, iDESC achieved the highest between-dataset consistency and validation rate based on genes found to be associated with IPF in public database.

## Materials and methods

### Statistical model

To identify cell type specific DE genes between two groups of subjects, iDESC uses a zero-inflated negative binomial mixed model to consider both subject effect and dropout events in scRNA-seq data with multiple subjects. The model includes two components: a zero component representing dropouts and a negative binomial component representing captured expression.

Suppose cells are collected from $n$ subjects. In a given cell type of interest, subject $i$ has ${m}_{i}$ cells so that there are in total $N={\sum }_{i=1}^{n}{m}_{i}$ cells of the given type. Let ${X}_{i}$ be the group label of subject $i$, where ${X}_{i}$ is 0 if subject $i$ belongs to group 1 and 1 if subject $i$ belongs to group 2. For each gene, let ${Y}_{ijk}$ denote the observed UMI count of gene $k$ in cell $j$ from subject $i$. We model the UMI count as:

$${Y}_{ijk}\left|{\pi }_{ijk},{\lambda }_{ik},{d}_{k}\right.\sim {\pi }_{ijk}\times {\mathbb{I}}_{\left\{{Y}_{ijk}=0\right\}}+\left(1-{\pi }_{ijk}\right)\times NB\left({S}_{ij}{\lambda }_{ik},{d}_{k}\right),$$

$$\mathrm{log}\left({\lambda }_{ik}\right)={\alpha }_{k}+{\beta }_{k}{X}_{i}+{\gamma }_{ik},$$

where ${\pi }_{ijk}$ is the dropout rate representing the probability of gene $k$ being dropped out in cell $j$ from subject $i$, ${\mathbb{I}}_{\left\{\cdot \right\}}$ is the indicator function that takes value 1 when the condition in the brackets is satisfied, 0 otherwise, ${S}_{ij}$ is the total UMI counts of cell $j$ from subject $i$, ${\lambda }_{ik}$ is the rate parameter of the negative binomial distribution representing the true underlying relative gene expression level, and ${d}_{k}$ is the dispersion parameter of gene $k$. The rate parameter ${\lambda }_{ik}$ is further modeled using a GLMM with log link, where ${\alpha }_{k}$ is the intercept, ${\beta }_{k}$ is the group effect representing the log fold change of mean expression of gene $k$ between the two groups, and ${\gamma }_{ik}$ is the gene-specific subject random effect, assumed to be independent and ${\gamma }_{ik} \sim N\left(0,{\sigma }_{k}^{2}\right)$.

Previous research found that the dropout rate of a given gene in a given cell depends on the expression level of the gene in the cell. Genes with lower expression level tend to have a higher dropout rate [16]. In addition, the dropout rates vary among cells and are influenced by the quality of sequencing library, cell type and RNA-seq protocol [16]. To quantify this dependency, we pooled information across genes and assumed that genes of similar average expression share similar dropout rates. In iDESC, we first calculated the proportion of zeros for each gene, and then fit a locally estimated scatterplot smoothing (LOESS) curve of the zero proportions against the log of gene-level average log-normalized UMI count across all cells. This overall LOESS curve captured the dependency of dropout rate on gene expression level and produced an initial estimate of dropout rates for all genes, denotes as ${\pi }_{k}^{0}$. Notice that the LOESS curve was obtained by assuming that the average log-normalized UMI count across all cells represents the true underlying gene expression. To relax this assumption, we introduce a parameter $\theta$ to allow the true dropout rate to deviate from the initial estimate when the single-cell gene expression is zero. Furthermore, the subject-level LOESS curves obtained from cells of the same subject have slight variations from the overall LOESS curve, suggesting that dropout rate is likely to be subject/batch specific. Putting together, we model the dropout rate ${\pi }_{ijk}$ as a GLMM with logit link:

$$\mathrm{logit}\left({\pi }_{ijk}\right)=\mathrm{logit}\left({\pi }_{k}^{0}\right)+\theta \times {\mathbb{I}}_{\left\{{Y}_{ijk}=0\right\}}+{\eta }_{ik},$$

where $\theta$ is the deviation from the initial estimate for a gene when its expression level is zero, and ${\eta }_{ik}$ is the gene-specific subject/batch random effect, assumed to be independent and ${\eta }_{ik} \sim N\left(0, {\tau }_{k}^{2}\right)$.

To test if gene $k$ is differentially expressed between the two groups, we constructed a Wald statistic to test ${H}_{0}: {\beta }_{k}=0$ against ${H}_{1}: {\beta }_{k}\ne 0$ using an R package ‘glmmTMB’ [61].

### Real datasets

We evaluated the performance of iDESC and other methods using three scRNA-seq datasets of whole lung samples from three independent IPF studies generated using the 10X Genomics Chromium platform. All datasets included IPF patients and healthy controls. In this article, we chose to focus on macrophage and fibroblast because both cell types have been recognized to play a significant role in IPF pathogenesis [43–50].

Kaminski refers to the scRNA-seq dataset of frozen distal lung parenchyma samples from 32 IPF and 28 control donor lungs in Adams et al. [18]. The raw data include 38,070 genes, 101,230 macrophages and 2,290 fibroblasts.

Kropski refers to the scRNA-seq dataset of fresh whole lungs from 10 IPF patients and 8 healthy donors in Habermann et al. [42]. The raw data include 31,054 genes, 11,532 macrophages and 1,644 fibroblasts.

Misharin refers to the scRNA-seq dataset of fresh lung tissues from 4 IPF patients and 8 transplant donors in Reyfman et al. [17]. The raw data include 21,807 genes, 8,534 macrophages and 2,468 fibroblasts.

### Data preprocessing

The raw UMI count matrices of all three datasets were downloaded from the links provided in the publications. We integrated the three datasets for data visualization and noticed that the cell type nomenclature in these three datasets were quite different. For example, the fibroblasts in the Kaminski dataset were distinguished by expressing *IGF*1 and *MFAP*5, and these cells do not express *ITGA*8 or *MYLK* as the myofibroblasts in the Kaminski dataset. However, a good portion of the fibroblasts in the Misharin dataset and the *PLIN*2+ fibroblasts in the Kropski dataset expressed *ITGA*8 and *MYLK*, suggesting that they are similar to the myofibroblasts in the Kaminski dataset but not the fibroblasts. The difference in cell type nomenclature may lead the DE analysis results to be invalidated across the three datasets. To ensure cells of the same type were compared across the three datasets so that DE analysis results were comparable, we conducted integration analysis across the three datasets using Seurat [62]. The graph-based Louvain clustering algorithm [63] was applied to cluster cells. Cell clusters with substantial overlap across the three datasets in the UMAP of integrated data were extracted for downstream DE analysis. Based on this nomenclature matching, we had 43,028 macrophages and 2,290 fibroblasts from the Kaminski dataset, 3,635 macrophages from the Misharin dataset, and 8,663 macrophages and 1,615 fibroblasts from the Kropski dataset. For each cell type, we filtered out genes that were expressed in less than 5% of cells and removed subjects with less than 5 cells. When choosing datasets to evaluate the between-dataset consistency of DE analysis results by each method, we selected datasets with a median number of cells per subject larger than 10 to ensure sample size in the analysis.

### Type I error assessment

To assess type I error, we randomly permuted the group labels of subjects in both Kaminski and Kropski datasets so that no model assumptions were made in data generation and the within-subject cell-to-cell correlation structure was preserved in the data. The permuted datasets were not expected to show transcriptomic difference between the two groups. We performed 500 permutations on each dataset and applied all DE analysis methods to the permuted datasets. Genes with a *p* value < 0.05 were considered significant. The empirical type I error for each gene was calculated as the proportion of permuted datasets having a *p* value < 0.05 for the given gene.

### Power comparison

To compare the statistical power of all methods, we simulated single-cell expression data with ground truth to mimic the real datasets. Macrophages in the Kaminski dataset were used for this analysis. First, for each gene, we fit the following ZINB model on the macrophage data from all subjects in the Kaminski dataset to estimate gene-level dispersion ${d}_{k}$, subject-specific dropout rate ${\pi }_{ik}$ and subject-specific relative gene expression level ${\lambda }_{ik}$: ${Y}_{ijk}\left|{\pi }_{ik},{\lambda }_{ik},{d}_{k}\right.\sim {\pi }_{ik}\times {\mathbb{I}}_{\left\{{Y}_{ijk}=0\right\}}+\left(1-{\pi }_{ik}\right)\times NB\left({S}_{ij}{\lambda }_{ik},{d}_{k}\right)$. The estimated ${\widehat{\pi }}_{ik}, {\widehat{\lambda }}_{ik}$ and ${\widehat{d}}_{k}$ were used in the simulation model to mimic real data. Second, we randomly sampled 30 subjects (15 IPF patients and 15 healthy controls) from the Kaminski dataset and extracted their ${\widehat{\pi }}_{ik}, {\widehat{\lambda }}_{ik}$ and ${\widehat{d}}_{k}$ to set up the simulation model. For each subject, $m$ macrophages ($m=20, 50, 100$) were simulated and their sequencing depths (${S}_{ij}$’s) were randomly sampled without replacement from the sequencing depths of all cells from the given subject. For each subject $i$, based on its parameter setting (${S}_{ij}$’s, ${\widehat{\pi }}_{ik}, {\widehat{\lambda }}_{ik}$ and ${\widehat{d}}_{k}$), two samples were simulated, one for each of the two groups. Let ${Y}_{ijk}^{1}$ and ${Y}_{ijk}^{2}$ be the simulated UMI counts of gene $k$ in cell $j$ for group 1 and group 2 samples generated from subject $i$, respectively, we have

$$\forall l\in \left\{1, 2\right\}: {Y}_{ijk}^{l}\sim {\pi }_{ijk}\times {\mathbb{I}}_{\left\{{Y}_{ijk}^{l}=0\right\}}+\left(1-{\pi }_{ijk}\right)\times NB\left({S}_{ij}{\widehat{\lambda }}_{ik}{e}^{{\beta }_{k}{\mathbb{I}}_{\left\{l=2\right\}}}, d\right),$$

$$\mathrm{logit}\left({\pi }_{ijk}\right)\sim N\left(\delta \times \mathrm{logit}\left({\widehat{\pi }}_{ik}\right), {\sigma }_{\pi }^{2}\right),$$

where $l$ is 1 for group 1 sample and 2 for group 2 sample, ${\beta }_{k}$ is the log fold change of gene $k$’s expression between the two groups, $d$ is the dispersion, $\delta$ is capture efficiency, and ${\sigma }_{\pi }^{2}$ is the variability of dropout rate. Based on the median of ${\widehat{d}}_{k}$ and the empirical estimation of variance of cell-level dropout rate, we set $d=1$ and ${\sigma }_{\pi }^{2}=2000$ in all simulations. To choose genes with moderate to high expression level, we randomly selected 450 genes with ${\widehat{\lambda }}_{ik}\ge \frac{1}{{S}_{ij}}$ for all cells and subjects, among which 150 were chosen to be DE genes and the remaining were non-DE genes. In each simulated dataset, for non-DE genes, the log fold change was set to ${\beta }_{k}=0$ and for the DE genes, ${\beta }_{k}$ was set to be the same value $\beta$, where $\beta$ varied from − 0.7 to − 0.1 and 0.1 to 0.7 with an increment of 0.1 across datasets. To simulate datasets with different levels of dropout rates, we set $\delta =0.5, 1, 1.5$, where the higher the capture efficiency is the fewer zeros exist in the simulated data.

We applied all methods to the simulated datasets to identify DE genes under different p-value threshold. By comparing the identified DE genes to the ground truth, the area under an ROC curve (AUC) score as well as the sensitivity and specificity under the p-value threshold of 0.05 were calculated for each method to evaluate method performance.

### scRNA-seq data analysis

We applied the DE analysis methods that consider subject effect to macrophage and fibroblast separately from the three real datasets. Method performance was assessed based on the consistency of the identified DE genes between datasets. Genes with a *p* value < 0.01 were considered to be differentially expressed. The cell-level effect coefficient [20] was used to demonstrate subject variation. To calculate the effect coefficient for each cell, we calculated dot products of log-normalized expression and the estimated group effects across the DE genes identified by iDESC and then scaled to a maximum absolute value of 1. To compare the consistency of DE genes between datasets, Fisher’s exact test was used to assess the significance of overlap and Jaccard index was calculated to measure the similarity between the two DE gene lists. For the overlapping DE genes between the two chosen datasets identified by each method in each cell type, we validated them using a list of 83 genes that are related to IPF in the Harmonizome database (https://maayanlab.cloud/Harmonizome/). The proportion of validated genes was calculated as the percentage of 83 genes that were the between-dataset overlapping DE genes.

### Supplementary Information

**Additional file 1: Figure S1.** Power comparison of iDESC and 7 methods without considering subject effect in simulated datasets. Evaluation criteria including (a) sensitivity and (b) specificity under the p-value threshold of 0.05, and (c) area under an ROC curve (AUC) to measure the accuracy of identified DE genes under three levels of capture efficiency (δ) and number of cells per subject (*m*). (d) ROC curves and the corresponding AUC scores when *β* = 0.4, *δ* = 1, *m* = 50.

**Additional file 2: Figure S2.** Boxplots showing the expression distribution of the top 3 DE genes, FN1, CCL18 and SPP1, in the three IPF macrophage datasets.

**Additional file 3: Table S1.** Runtime of five methods using a single core.

## Data Availability

All analyses were run in R v3.5.3. An R package implementing the proposed method is available at https://github.com/yl883/iDESC under the MIT license and was deposited to Zenodo (https://doi.org/10.5281/zenodo.6929851). The description of used data sets is in the “Real datasets” section. Barcodes of cells used for analysis can be found at Zenodo.

## Abbreviations

scRNA-seq: Single-cell RNA sequencing
UMI: Unique Molecular Identifier
IPF: Idiopathic pulmonary fibrosis
DMSO: Dimethyl sulfoxide
ROC: Receiver operating characteristic
AUC: Area under the curve
