# Correction: iDESC: identifying differential expression in single-cell RNA sequencing data with multiple subjects

**DOI:** 10.1186/s12859-023-05523-6

**Published:** 2023-10-19

**Authors:** Yunqing Liu, Jiayi Zhao, Taylor S. Adams, Ningya Wang, Jonas C. Schupp, Weimiao Wu, John E. McDonough, Geoffrey L. Chupp, Naftali Kaminski, Zuoheng Wang, Xiting Yan

**Affiliations:** 1 Department of Biostatistics, Yale School of Public Health, New Haven, CT 06520 USA; 2 Section of Pulmonary, Critical Care and Sleep Medicine, Yale School of Medicine, New Haven, CT 06520 USA; 3 Department of Respiratory Medicine, Hannover Medical School and Biomedical Research in End-Stage and Obstructive Lung Disease Hannover, German Center for Lung Research (DZL), Hannover, Germany; 4 Meta Platforms, Inc, Cambridge, USA

**Correction: BMC Bioinformatics (2023) 24:318** 10.1186/s12859-023-05432-8

Following publication of the original article [1], the authors identified an error in Table 2. The correct table is given below.

The incorrect Table 2 is:

**Table 2:** Fisher’s exact test and Jaccard index measuring the DE genes overlapping between the two chosen scRNA-seq datasets in macrophage and fibroblast

Method	iDESC	MAST-RE	Muscat-MM	Muscat-PB	subT
Macrophage					
*P* value	$1\times {10}^{-5}$	$1.000$	$0.269$	$1\times {10}^{-5}$	0.085
Jaccard Index	0.137	$0.061$	$0.108$	0.055	0.028
Fibroblast					
*P* value	$4\times {10}^{-21}$	$4\times {10}^{-6}$	$3\times {10}^{-5}$	$2\times {10}^{-7}$	0.011
Jaccard Index	0.077	0.067	0.071	0.035	0.024

The correct Table 2 is:

**Table 2:** Fisher’s exact test and Jaccard index measuring the DE genes overlapping between the two chosen scRNA-seq datasets in macrophage and fibroblast

Method		iDESC	MAST-RE	Muscat-MM	Muscat-PB	subT
Macrophage	P-value	$1\times {10}^{-5}$	$1.000$	$0.269$	$1\times {10}^{-5}$	$0.085$
	Jaccard Index	0.137	$0.061$	$0.108$	0.055	$0.028$
Fibroblast	P-value	$4\times {10}^{-21}$	$4\times {10}^{-6}$	$3\times {10}^{-5}$	$2\times {10}^{-7}$	$0.011$
	Jaccard Index	0.077	0.067	0.071	0.035	0.024
