# Supplementary figures and images for: iDESC: identifying differential expression in single-cell RNA sequencing data with multiple subjects
Source: BMC Bioinformatics. 2023 Aug 22;24:318. doi: 10.1186/s12859-023-05432-8 (PMC10463720; doi:10.1186/s12859-023-05432-8)

**a**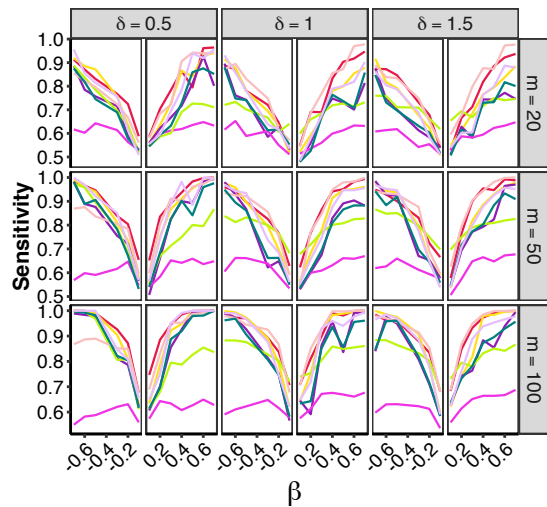**b**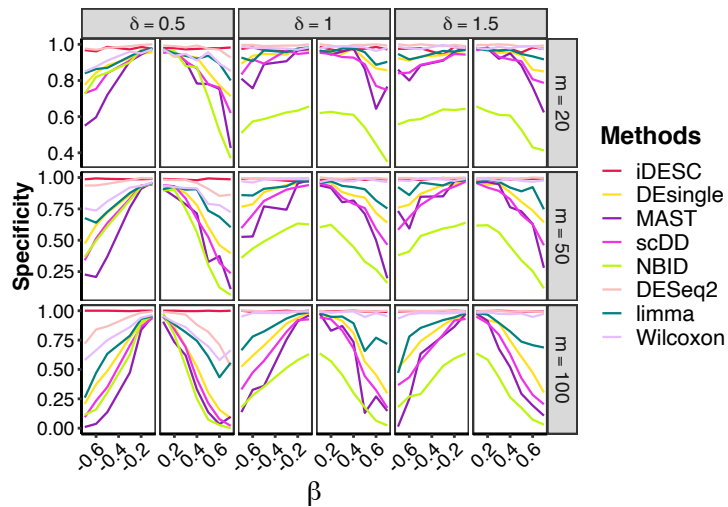**c**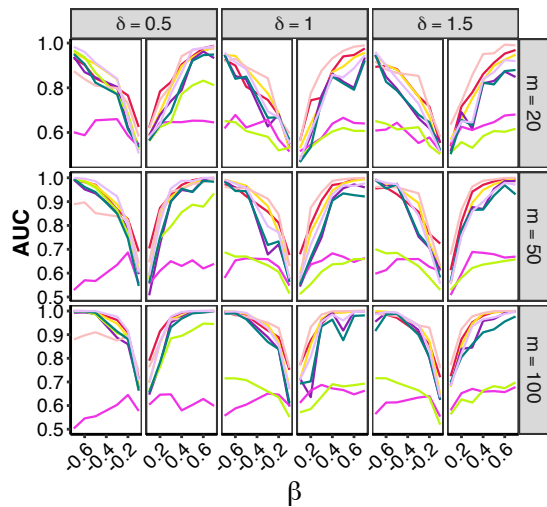**d**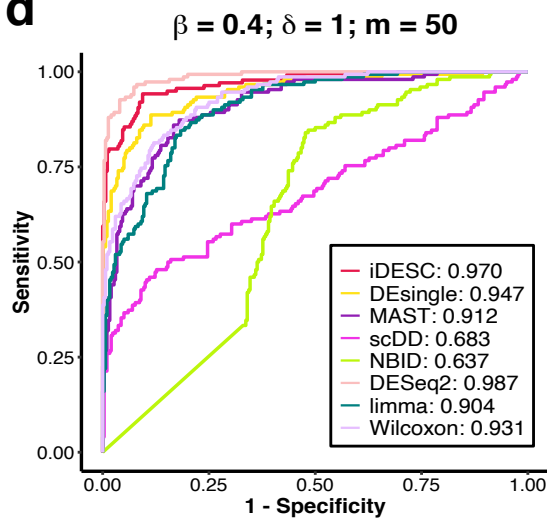

Supplement: Supplementary file 1 — Additional file 1: Figure S1. Power comparison of iDESC and 7 methods without considering subject effect in simulated datasets. Evaluation criteria including (a) sensitivity and (b) specificity under the p-value threshold of 0.05, and (c) area under an ROC curve (AUC) to measure the accuracy of identified DE genes under three levels of capture efficiency (δ) and number of cells per subject (m). (d) ROC curves and the corresponding AUC scores when β = 0.4, δ = 1, m = 50. [file 12859_2023_5432_MOESM1_ESM.pdf]

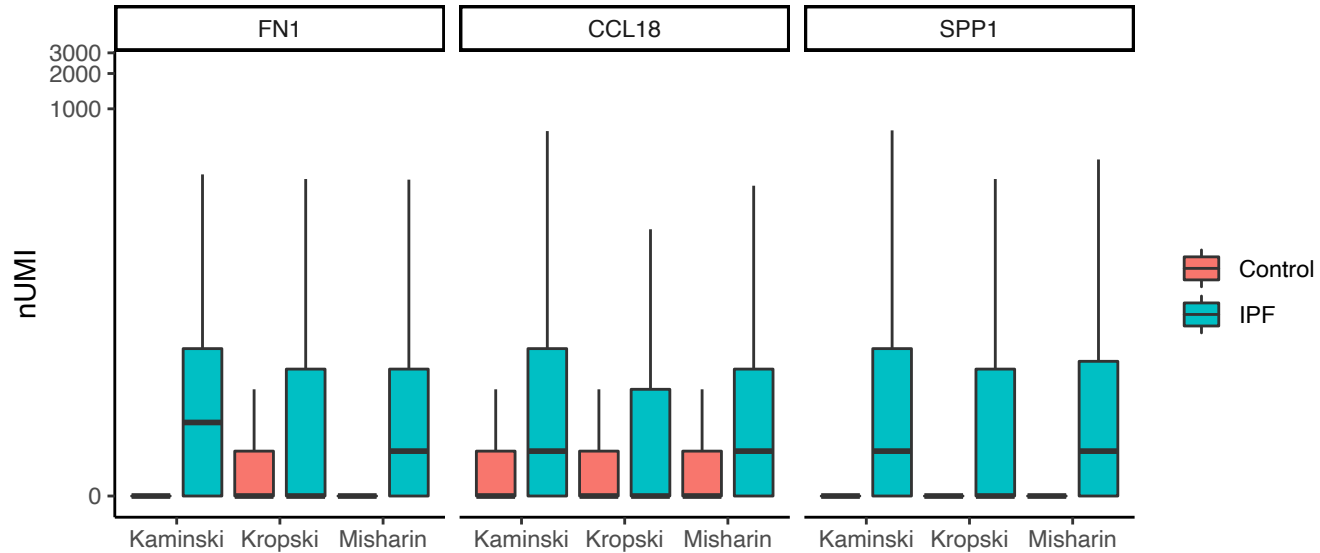

Supplement: Supplementary file 2 — Additional file 2: Figure S2. Boxplots showing the expression distribution of the top 3 DE genes, FN1, CCL18 and SPP1, in the three IPF macrophage datasets. [file 12859_2023_5432_MOESM2_ESM.pdf]
