# Supplementary material for: iDESC: identifying differential expression in single-cell RNA sequencing data with multiple subjects
Source: BMC Bioinformatics. 2023 Aug 22;24:318. doi: 10.1186/s12859-023-05432-8 (PMC10463720; doi:10.1186/s12859-023-05432-8)
Supplement: Supplementary file 3 — Additional file 3: Table S1. Runtime of five methods using a single core. [file 12859_2023_5432_MOESM3_ESM.docx]

**Table S1.** Runtime of five methods using a single core.

| **Method** | **iDESC** | **MAST-RE** | **Muscat-MM** | **Muscat-PB** | **subT** |
| --- | --- | --- | --- | --- | --- |
| **Runtime (s)** | 95.25 | 8.08 | 94.52 | 1.21 | 0.22 |
